# Supplementary figures and images for: Tools and pipelines for BioNano data: molecule assembly pipeline and FASTA super scaffolding tool
Source: BMC Genomics. 2015 Sep 29;16:734. doi: 10.1186/s12864-015-1911-8 (PMC4587741; doi:10.1186/s12864-015-1911-8)

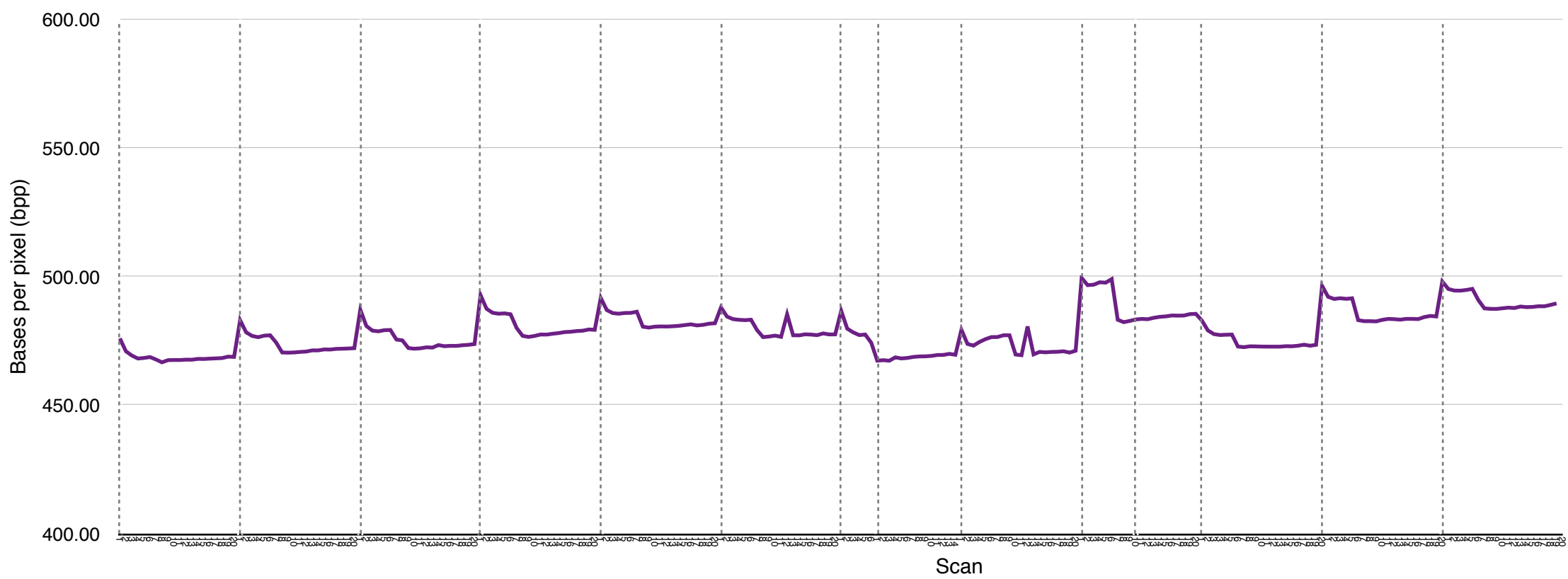

Supplement: Additional file 1 — Single molecule map stretch per scan in recent flowcells. Bases per pixel (bpp) is plotted for scans 1..n for each flowcell of mouse lemur molecules (purple). The first scan of each flowcell is indicated with a grey dashed line. The pre-adjusted molecule map stretch was determined by aligning molecule maps to the in silico maps. Data made available by P.A. Larsen, J. Rogers, A.D. Yoder and the Duke Lemur Center. (ZIP 55 kb) [file 12864_2015_1911_MOESM1_ESM.zip › Supplemental_1_bpp_per_scan.pdf]
