# Supplementary material for: Tools and pipelines for BioNano data: molecule assembly pipeline and FASTA super scaffolding tool
Source: BMC Genomics. 2015 Sep 29;16:734. doi: 10.1186/s12864-015-1911-8 (PMC4587741; doi:10.1186/s12864-015-1911-8)
Supplement: Additional file 3 — Single molecule map metrics and histograms from T. castaneum DNA. Detailed metrics for molecule maps including map N50, cumulative length and number of maps. Figures show histograms of per molecule map quality metrics including length, molecule map SNR and intensity, label count, label SNR and label intensity. Molecule maps are filter for minimum molecule lengths of 100, 150 or 180 kb. (ZIP 39.8 kb) [file 12864_2015_1911_MOESM3_ESM.zip › Supplemental_3_MapStatsHistograms_min_length_180kb.pdf]

Summary metrics for molecule maps > 180 kb within all BNX files

Molecule map N50: 232.566 (kb)

Cumulative length of molecule maps: 34287.1472702 (Mb)

Number of molecule maps: 139949

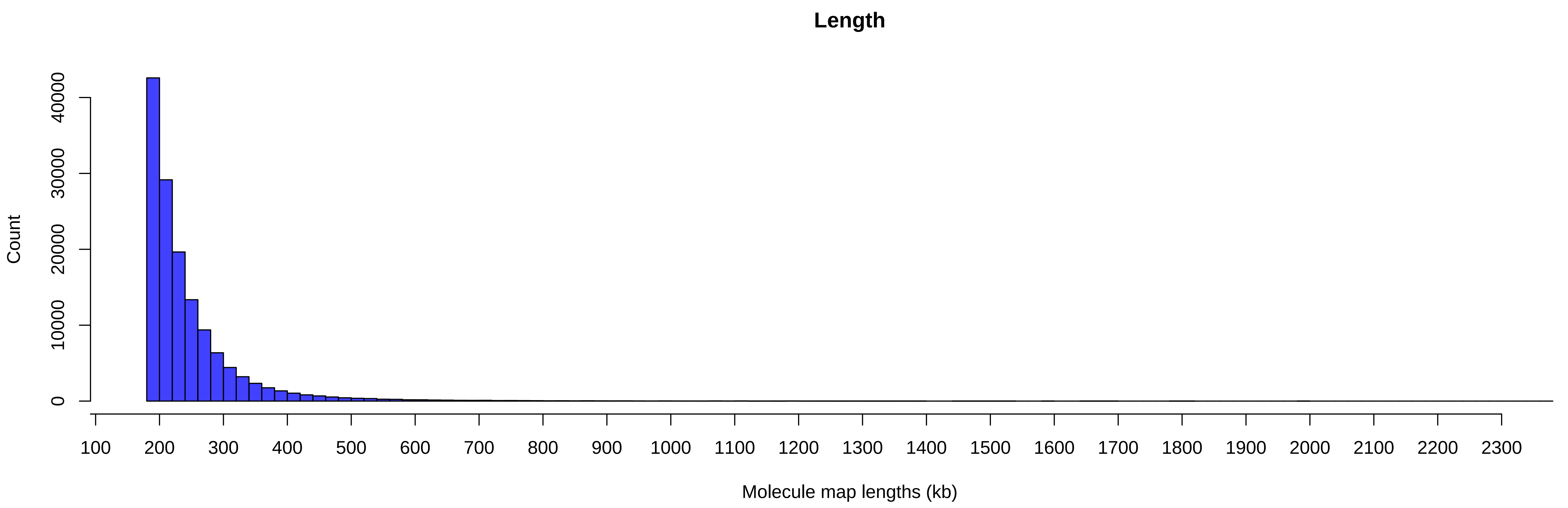
Length
Count
0
10000
20000
30000
40000
100
200
300
400
500
600
700
800
900
1000
1100
1200
1300
1400
1500
1600
1700
1800
1900
2000
2100
2200
2300
Molecule map lengths (kb)

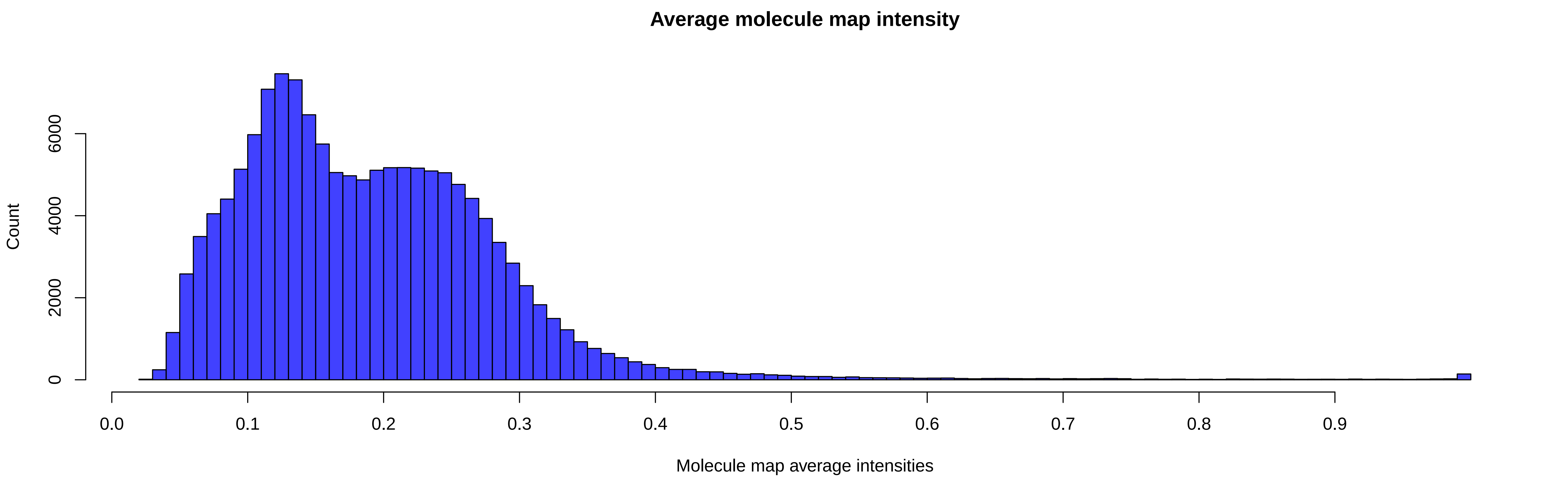

Average molecule map intensity
Count
0
2000
4000
6000
0.0
0.1
0.2
0.3
0.4
0.5
0.6
0.7
0.8
0.9
Molecule map average intensities

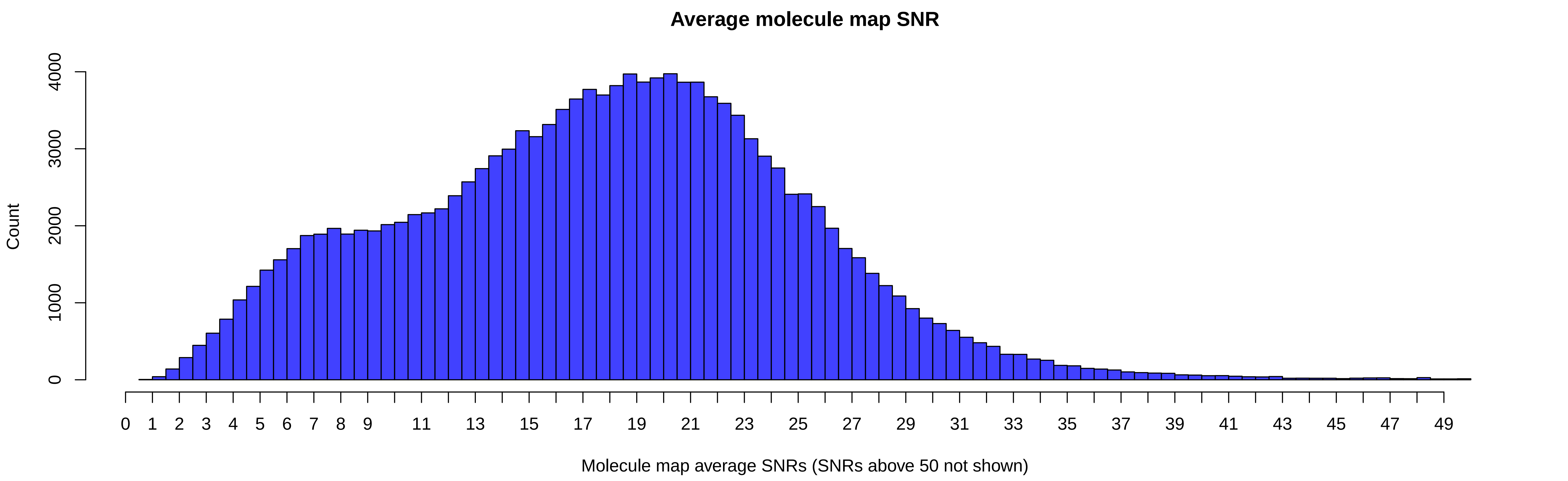
Average molecule map SNR
Count
0
1000
2000
3000
4000
0 1 2 3 4 5 6 7 8 9 11 13 15 17 19 21 23 25 27 29 31 33 35 37 39 41 43 45 47 49
Molecule map average SNRs (SNRs above 50 not shown)

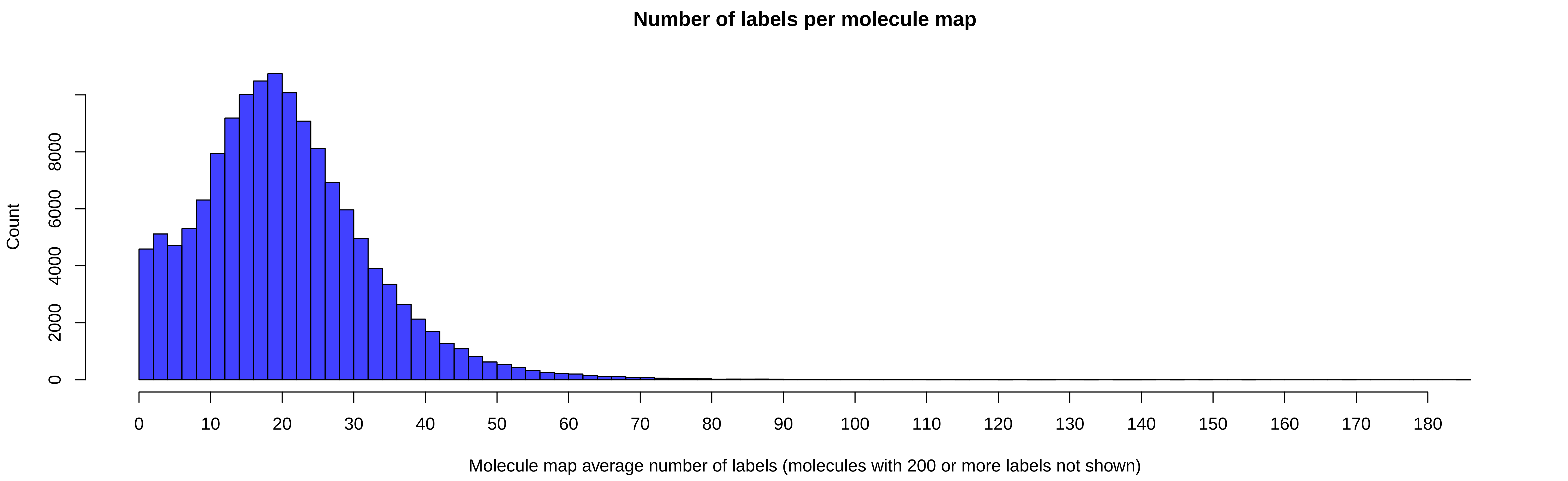
Number of labels per molecule map
Count
0
2000
4000
6000
8000
0
10
20
30
40
50
60
70
80
90
100
110
120
130
140
150
160
170
180
Molecule map average number of labels (molecules with 200 or more labels not shown)

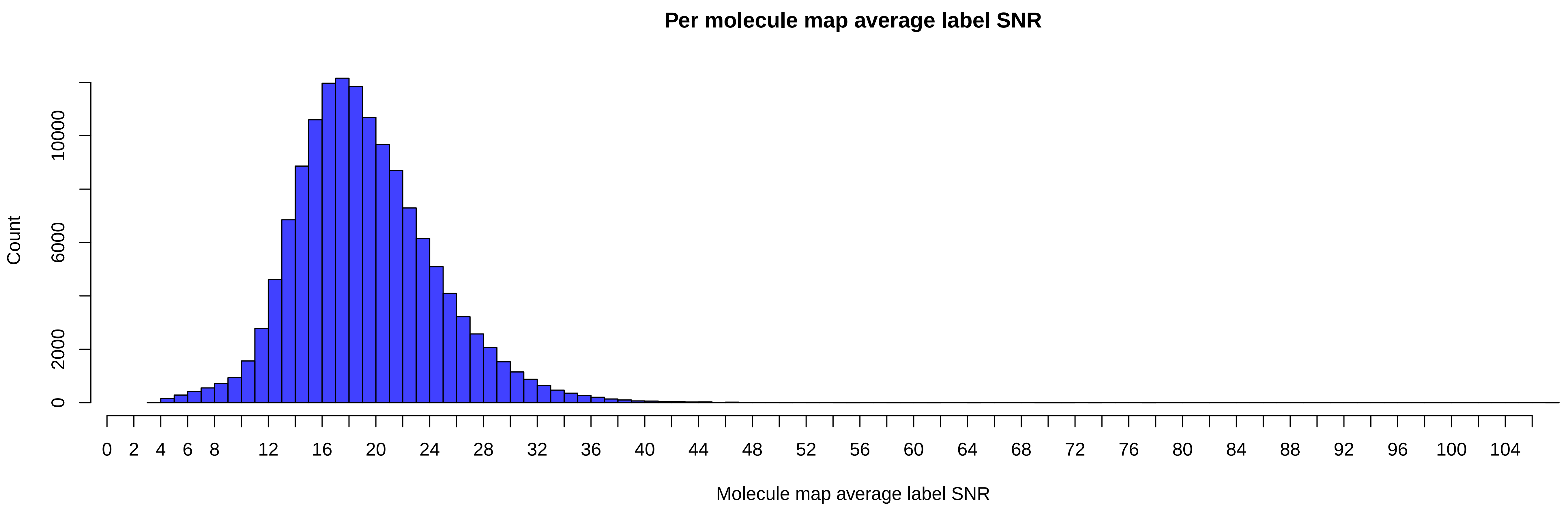

Per molecule map average label SNR
Count
0
2000
6000
10000
0 2 4 6 8 12 16 20 24 28 32 36 40 44 48 52 56 60 64 68 72 76 80 84 88 92 96 100 104
Molecule map average label SNR

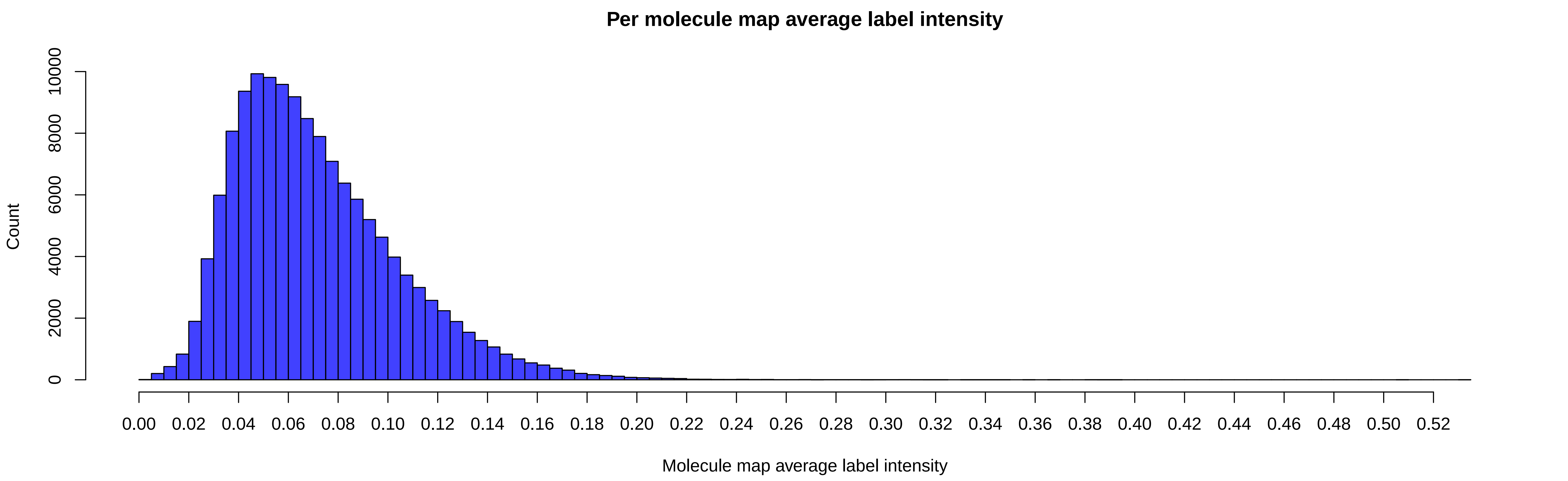
Per molecule map average label intensity
Count
0
2000
4000
6000
8000
10000
0.00 0.02 0.04 0.06 0.08 0.10 0.12 0.14 0.16 0.18 0.20 0.22 0.24 0.26 0.28 0.30 0.32 0.34 0.36 0.38 0.40 0.42 0.44 0.46 0.48 0.50 0.52
Molecule map average label intensity
