# Supplementary material for: Tools and pipelines for BioNano data: molecule assembly pipeline and FASTA super scaffolding tool
Source: BMC Genomics. 2015 Sep 29;16:734. doi: 10.1186/s12864-015-1911-8 (PMC4587741; doi:10.1186/s12864-015-1911-8)
Supplement: Additional file 5 — ChLGs before and after super scaffolding. Alignments of Tcas5.0 and Tcas5.2 in silico maps to consensus genome maps for all ChLGs. Consensus genome maps (blue with molecule coverage shown in dark blue) aligned to the in silico maps (green with contigs overlaid as translucent colored squares). Alignment to both Tcas5.2 super scaffolds (top alignment) and Tcas5.0 scaffolds (bottom alignment) are shown. (PDF 408 kb) [file 12864_2015_1911_MOESM5_ESM.pdf]

Additional file 5

Jennifer Shelton[1], Cassondra Coleman[2], Nic Herndon[3], Nanyan Lu[4], Ernest Lam[5], Thomas Anantharaman[6], Palak Sheth[7], and Sue Brown[8]

[1]*Kansas State University*
[2]*Affiliation not available*
[3]*Affiliation not available*
[4]*Affiliation not available*
[5]*Affiliation not available*
[6]*Affiliation not available*
[7]*Affiliation not available*
[8]*Affiliation not available*

June 11, 2015

Abstract

Additional file 5 — ChLGs before and after super scaffolding Alignments of Tcas5.0 and Tcas5.2 *in silico* maps to consensus genome maps for all ChLGs. Consensus genome maps (blue with molecule coverage shown in dark blue) aligned to the *in silico* maps (green with contigs overlaid as translucent colored squares). Alignment to both Tcas5.2 super scaffolds (top alignment) and Tcas5.0 scaffolds (bottom alignment) are shown.

Competing interests

JMS, MCC, NH, NL, and SJB declare that they have no competing interests. ETL, PS and TA are employees at BioNano Genomics and hold stock options.

Figures

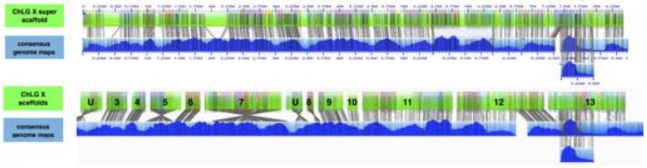

Figure 1: **Chromosome Linkage Group X.** ChLGX before and after super scaffolding viewed in IrysView.

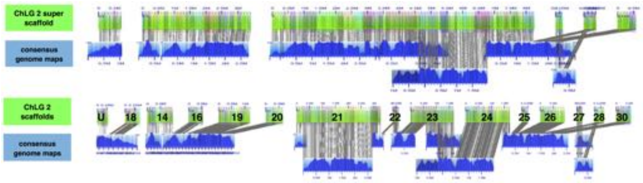

Figure 2: **Chromosome Linkage Group 2.** ChLG2 before and after super scaffolding viewed in IrysView.

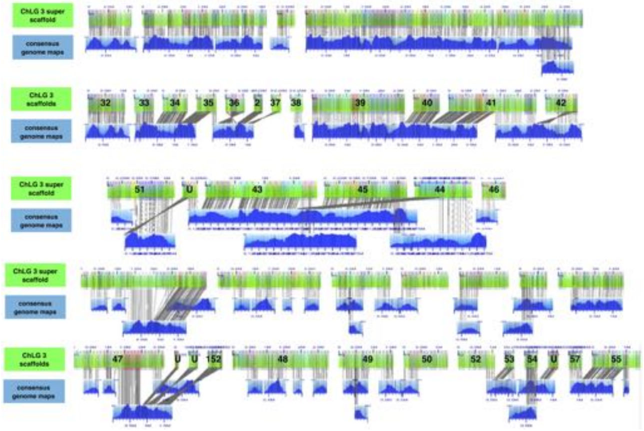

Figure 3: **Chromosome Linkage Group 3.** ChLG3 before and after super scaffolding viewed in IrysView.

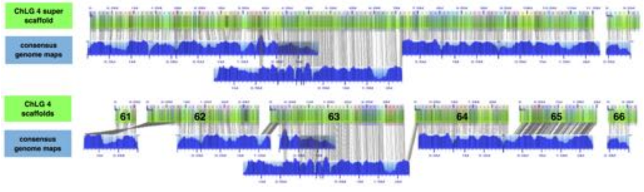

Figure 4: **Chromosome Linkage Group 4.** ChLG4 before and after super scaffolding viewed in IrysView.

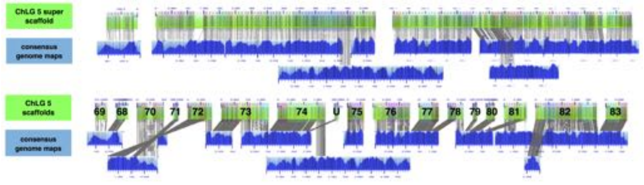

Figure 5: **Chromosome Linkage Group 5.** ChLG5 before and after super scaffolding viewed in IrysView.

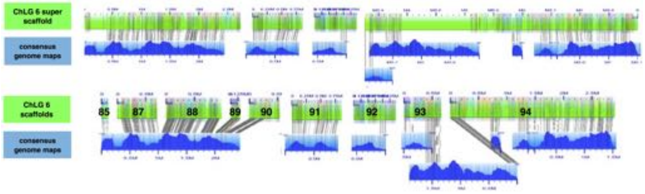

Figure 6: **Chromosome Linkage Group 6.** ChLG6 before and after super scaffolding viewed in IrysView.

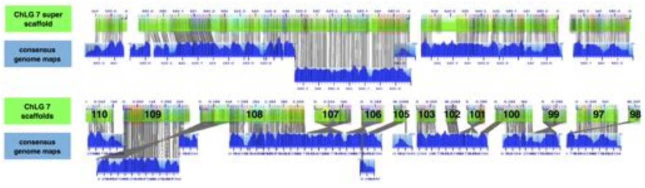

Figure 7: **Chromosome Linkage Group 7.** ChLG7 before and after super scaffolding viewed in IrysView.

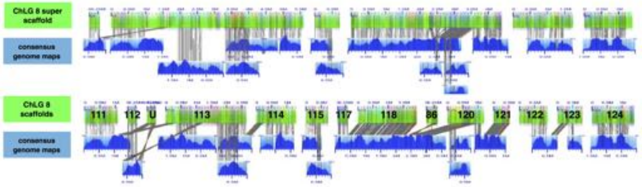

Figure 8: **Chromosome Linkage Group 8.** ChLG8 before and after super scaffolding viewed in IrysView.

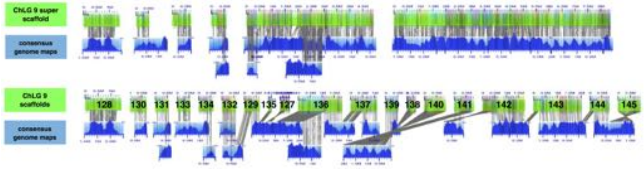

Figure 9: **Chromosome Linkage Group 9.** ChLG9 before and after super scaffolding viewed in IrysView.

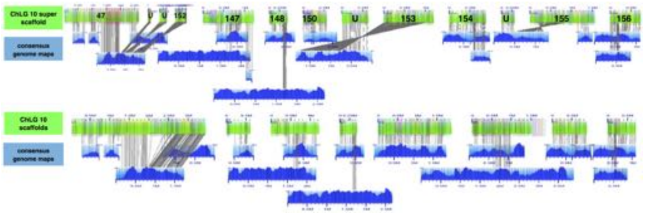

Figure 10: **Chromosome Linkage Group 10.** ChLG10 before and after super scaffolding viewed in IrysView.
