# Supplementary material for: Tools and pipelines for BioNano data: molecule assembly pipeline and FASTA super scaffolding tool
Source: BMC Genomics. 2015 Sep 29;16:734. doi: 10.1186/s12864-015-1911-8 (PMC4587741; doi:10.1186/s12864-015-1911-8)
Supplement: Additional file 6 — Assembly and super scaffolding with multiple genera. We examined experiments from 16 different genera to determine if the results seen for the Tribolium castaneum genome are typical for other genomes as well. (PDF 876 kb) [file 12864_2015_1911_MOESM6_ESM.pdf]

Additional file 6

Jennifer Shelton[1], Cassondra Coleman[2], Nic Herndon[3], Nanyan Lu[4], Ernest Lam[5], Thomas Anantharaman[6], Palak Sheth[7], and Sue Brown[8]

[1]*Kansas State University*
[2]*Affiliation not available*
[3]*Affiliation not available*
[4]*Affiliation not available*
[5]*Affiliation not available*
[6]*Affiliation not available*
[7]*Affiliation not available*
[8]*Affiliation not available*

June 11, 2015

Abstract

Assembly and super scaffolding with multiple genera.

We examined experiments from 16 different genera to determine if the results seen for the *Tribolium castaneum* genome are typical for other genomes as well. The *T. castaneum* genome map N50 was found to be in the high end of the probability density distribution (Additional file 6; Figure 1). The same is true for the Tcas5.0 draft sequence assembly N50 and percent of N50 improvement after super scaffolding compared to the other 17 of 19 total projects that had draft sequence genomes (Additional file 6; Figure 1). However, in no case was the *T. castaneum* value the highest value recorded, suggesting that a wide range of output quality is possible including values better and worse than the output for *T. castaneum*.

We checked for evidence of correlations between a range of genomic metrics and map assembly, alignment or FASTA super scaffolding results. Because many of the genomic metrics had very broad ranges with variance that increased often for higher values the genomic metrics were log transformed to compress the upper tails and stretch the lower tails of the distributions.

Overall we found little correlation between either sequence FASTA N50, molecule map coverage or molecule map label density and final genome map N50. We did, however, find correlations between finished map and sequence assembly metrics and alignment and super scaffolding quality. There is a positive correlation between high value sequence

assembly metrics and *in silico* map-to-genome map alignment metrics (Additional file 6; Figures 3-5) as well as post super scaffolding N50 improvement (Additional file 6; Figures 3,5). There is also a positive correlation between high value genome map assembly metrics and post super scaffolding N50 improvement (Additional file 6; Figures 3,5). However, no direct correlation was found between sequence assembly N50 and genome map N50 (Additional file 6; Figures 4-5). Taken together the analysis suggests different factors may determine sequence assembly and genome map assembly quality. Although sequence assembly N50 may not be useful to predict genome map N50, if both independent assemblies have high N50's than more of the map lengths may align and super scaffolding may be more productive.

The low degree of correlation found between genome map N50 and sequence N50 may stem from steps unique to the molecule map imaging process. It might be expected that a genome with sequence that assembles well may have qualities that would also favor molecule map assembly (e.g. low repeat content, low ploidy, inbreed lines, etc.). However molecule map assembly is also influenced by unique factors like frequency of fragile sites (two labels occurring on opposite strands in close proximity), labeling efficiency and ability to extract high molecular weight DNA all of which vary for different organisms.

Principal component analysis suggests a negative correlation between labels per 100 kb and molecule coverage (Additional file 6: Figures 2-3). The correlation between labels per 100 kb and molecule coverage was weakly significant in individual regression (Additional file 6; Figures 4-5). Labels per 100 kb are monitored as molecules are being imaged. Lower than expected label density can occasionally lead to further labeling reactions or other adjustments to data collection and therefore greater depth of coverage.

Overall, comparison of the results for the *T. castaneum* genome and 19 additional genome projects suggest that results may vary widely from project to project. Many factors may contribute to this effect including the quality of the sequence assembly, degree of divergence between the organism or organisms used to extract DNA, success of extraction and labeling of high molecular weight DNA, genome size and genome complexity. In fact, the tendency for assemblies from the same genera or species to cluster together on the PCA plots suggests that organism-specific qualities may influence assembly, alignment or super scaffolding results (Additional file 6: Figures 2-3). Although analysis of more projects is needed to determine if these similarities are meaningful predictors of output quality.

Competing interests

The JMS, MCC, NH, NL, and SJB declare that they have no competing interests. ETL, PS and TA are employees at BioNano Genomics and hold stock options.

Acknowledgements

Matthias Weissensteiner Jochen Wolf, Uppsala University. Stephen Schaeffer from The Pennsylvania State University and Stephen Richards from the Baylor College of Medicine Human Genome Sequencing Center for the use of the *D. pseudoobscura* data. Mike Kanost from Kansas State University. Jeff Maughan from Brigham Young University for the use of the *Amaranth* data. The Udall Lab from Brigham Young University and Cotton Inc. for the use of the cotton data. Grant (NSF 1237993) for use of the *Medicago* data. Christopher Cunningham, University of Georgia for the use of Nicrophorus data. Catherine Peichel from the Fred Hutchinson Cancer Research Center and Michael White from the University of Georgia for the *Gasterosteus* data. Mirkó Palla, Ph.D., Wyss Institute Postdoctoral Fellow, Church; Laboratory - Department of Genetics, Harvard Medical School and George Church, Ph.D., Wyss Institute Core Faculty Member, Robert Winthrop Professor of Genetics at Harvard Medical School, Professor of Health Sciences and Technology at Harvard and MIT, and Senior Associate Member at the Broad Institute of Harvard and MIT for the *Escherichia coli* data.

Figures

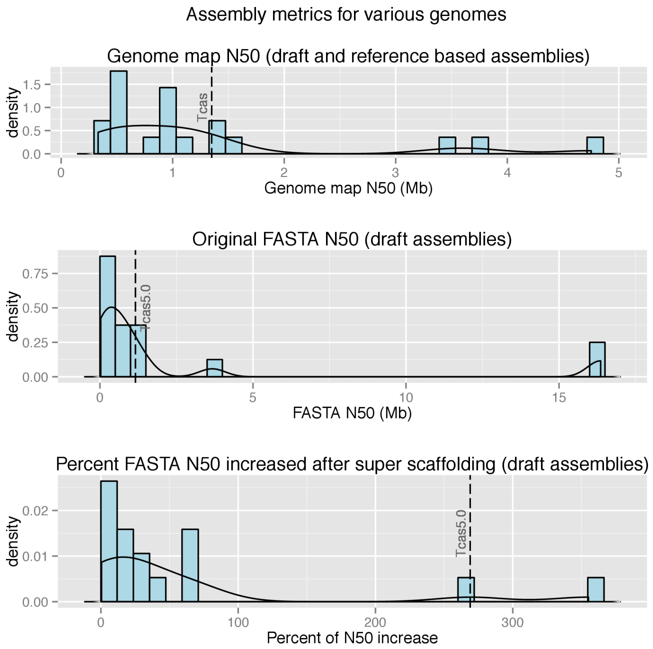

Figure 1: **Assembly metrics for various genomes.** Raw assembly metrics are plotted along the x-axis with density on the y-axis. Plots include a density histogram (blue) and a smooth density estimate (black line). The x-axis value for the Tcas5.0 draft assembly is also indicated (dashed black line). In all plots the Tcas5.0 draft assembly or the Tcas genome maps are on the higher end of the distribution indicating that the initial draft assembly was relatively high quality and that the results of molecule map assembly and super scaffolding were also relatively high quality. In no plot was the Tcas5.0 draft assembly in the highest value bin.

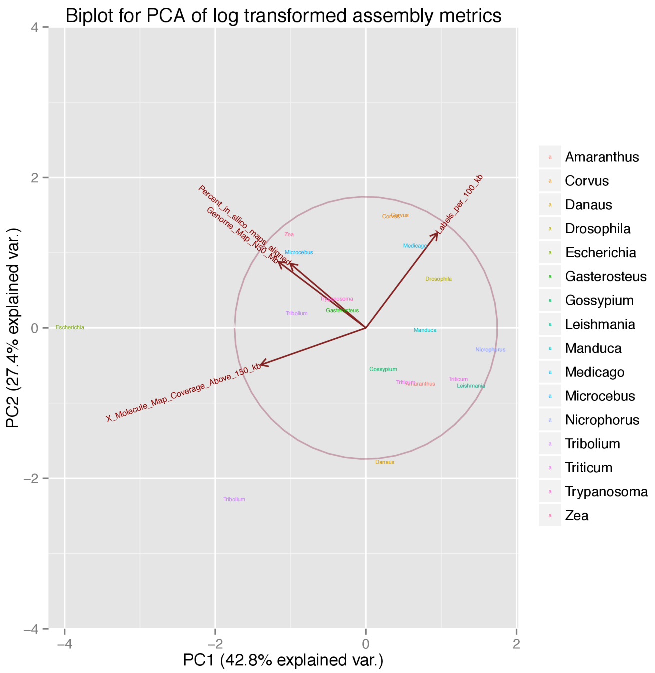

Figure 2: **Biplot of the first and second principal components for the log transformed assembly metrics.** Multicolored samples representing individual assemblies are identified by genus. The assembly metrics included as vectors are indicated in burgundy. PC1 explained 42.8% of the variance in the included studies. Genome map N50, percent of length of *in silico* map aligned with genome maps and molecule map coverage were positively correlated with each other and negatively correlated with molecule map labels per 100 kb. If higher label density increased the number of fragile sites within the genome this could negatively effect the proportion of the molecules that remain long enough for molecule length filters and could impede assembly across fragile sites in genome maps. PC2 explained 27.4% of the variance. Primarily in PC2 genome map N50, percent of length of *in silico* map aligned with genome maps and molecule map labels per 100 kb were positively correlated with each other and negatively correlated with molecule map coverage. Overall, genome map N50 and percent of length of *in silico* map aligned with genome maps were positively correlated with each other in both PC1 and PC2. Because many of the genomic metrics had very broad ranges with variance that increased often for higher values the genomic metrics were log transformed to compress the upper tails and stretch the lower tails.

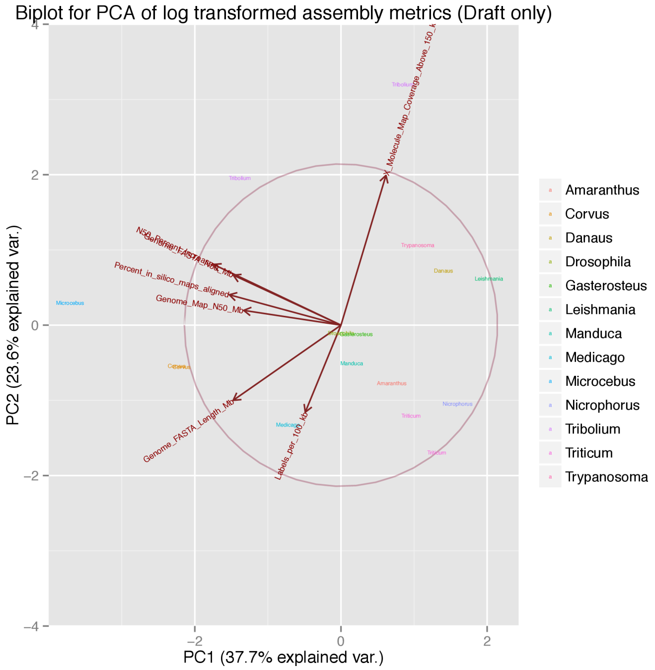

Figure 3: **Biplot of the first and second principal components for the log transformed assembly metrics (Draft only).** Multicolored samples representing individual assemblies are identified by genus. The assembly metrics included as vectors are indicated in burgundy. PC1 explained 37.7% of the variance in the included studies. In PC1, FASTA N50, genome map N50, increase in FASTA N50 after super scaffolding, percent of *in silico* map length aligned to genome maps all showed a strong positive correlation to each other. Genome FASTA length also showed a positive correlation with these variables. PC2 explained 23.6% of the variance. In this PC molecule map coverage is strongly negatively correlated with molecule map labels per 100 kb and genome FASTA length. Labels per 100 kb values are monitored as data is collected and compared to estimated label density. Lower than expected label density can occasionally lead to further labeling reactions or other adjustments to data collection and therefore greater depth of coverage. This may be more common for unsupported samples (i.e. a species that has not previously been used to create molecule maps). Larger genomes take more data collection to provide the same fold coverage of the genome. Therefore it is not surprising that FASTA length is negatively correlated with molecule map coverage depth. Because many of the genomic metrics had very broad ranges with variance that increased often for higher values the genomic metrics were log transformed to compress the upper tails and stretch the lower tails.

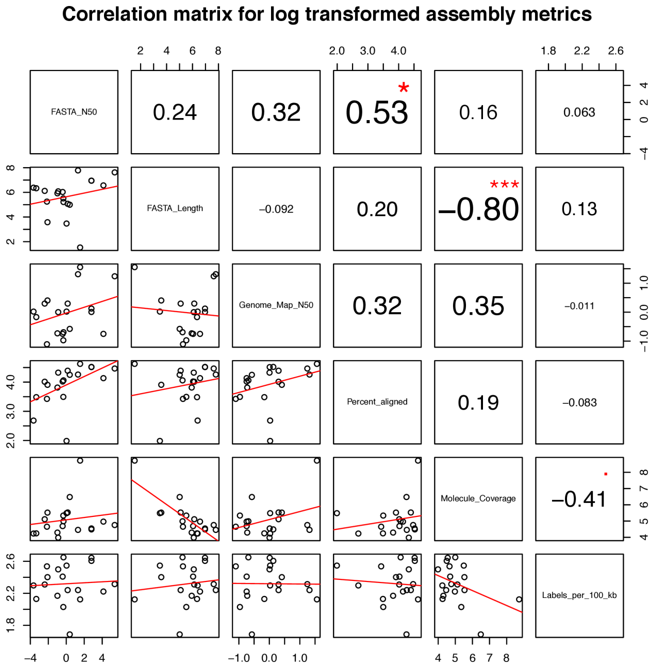

Interestingly, there was no significant correlation indicated between genome map N50 and sequence assembly N50. One might expect that a genome with sequence that assembles easily may have qualities that would also favor molecule map assembly (e.g. low repeat content, low ploidy, inbreed lines, etc.). However molecule assembly is also influenced by unique factors like frequency of fragile sites (two labels occurring on opposite strands in close proximity), labeling efficiency and ability to extract high molecular weight DNA all of which vary for different organisms. The label density, labels per 100 kb of molecule maps, had a weakly significant negative correlation with molecule coverage (-0.41, $p = 0.078$). Labels per 100 kb of molecule maps had no significant correlation with any other single genomic metric. Because many of the genomic metrics had very broad ranges with variance that increased often for higher values the genomic metrics were log transformed to compress the upper tails and stretch the lower tails.

Interestingly, there was no significant correlation indicated between genome map N50 and sequence assembly N50. One might expect that a genome with sequence that assembles easily may have qualities that would also favor molecule map assembly (e.g. low repeat content, low ploidy, inbreed lines, etc.). However molecule assembly is also influenced by unique factors like frequency of fragile sites (two labels occurring on opposite strands in close proximity), labeling efficiency and ability to extract high molecular weight DNA all of which vary for different organisms. The label density, labels per 100 kb of molecule maps, had a weakly significant negative correlation with molecule coverage (-0.41, $p = 0.078$). Labels per 100 kb of molecule maps had no significant correlation with any other single genomic metric. Because many of the genomic metrics had very broad ranges with variance that increased often for higher values the genomic metrics were log transformed to compress the upper tails and stretch the lower tails.

Figure 4: **Correlation matrix for log transformed assembly metrics.** Diagonal panels indicate the assembly metric used as the x-value in the respective column and the y-value in the respective row. Lower panels show XY scatter plots of each metric against all other metrics with a best fit line (red). Upper panels show the correlation coefficient (with font scaled based on the absolute value of the correlation coefficient). Significance of correlation coefficient is indicated in red (where "" means $p < 1$, "." means $p < 0.1$, "*" means $p < 0.05$, [illegible]

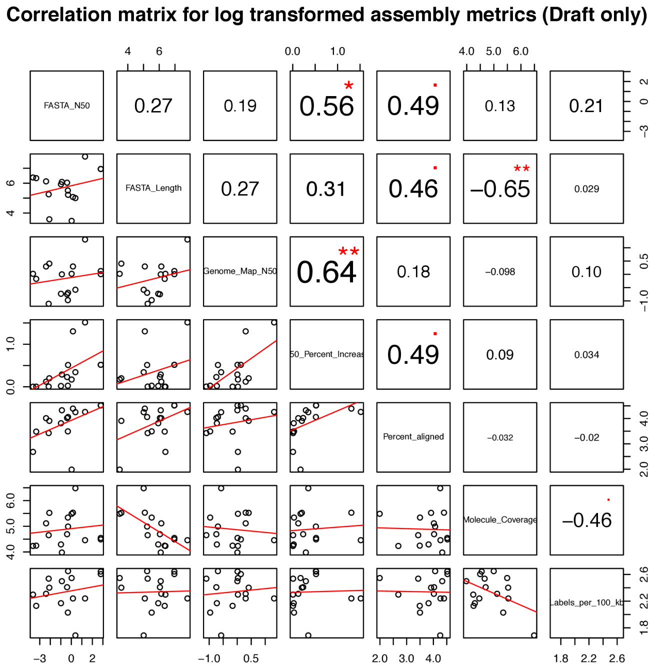

Figure 5: **Correlation matrix for log transformed assembly metrics (Draft only).** Diagonal panels indicate the assembly metric used as the x-value in the respective column and the y-value in the respective row. Lower panels show XY scatter plots of each metric against all other metrics with a best fit line (red). Upper panels show the correlation coefficient (with font scaled based on the absolute value of the correlation coefficient). Significance of correlation coefficient is indicated in red (where "" means $p < 1$, "." means $p < 0.1$, "*" means $p < 0.05$, "**" means $p < 0.01$ and "***" means $p < 0.001$). Again FASTA N50 positively correlated with the percent of the total length of *in silico* maps to align to genome maps (0.49, $p = 0.052$). FASTA length positively correlated with the percent of the total length of *in silico* maps to align to genome maps (0.46, $p = 0.076$). The percent increase in FASTA N50 after super scaffolding with Stitch significantly positively correlated with genome map N50 and FASTA N50 (0.64, $p = 0.007$; 0.56, $p = 0.023$). For the projects with draft sequence assemblies there was no significant correlation between molecule map coverage and genome map N50. There was still a significant negative correlation between FASTA length and molecule map coverage (-0.65, $p = 0.007$). Again, the label density, labels per 100 kb of molecule maps, had a weakly significant negative correlation with molecule coverage (-0.46, $p = 0.075$). Also, labels per 100 kb of molecule maps had no significant correlation with any other single genomic metric. Because many of the genomic metrics had very broad ranges with variance that increased often for higher values the genomic metrics were log transformed to compress the upper tails and stretch the lower tails.
